# Central sensitization and pain hypersensitivity: Some critical considerations.

**DOI:** 10.12688/f1000research.15956.2

**Published:** 2018-08-31

**Authors:** Emanuel N. van den Broeke

**Affiliations:** 1 Institute of Neuroscience, Division Systems and Cognition, Universite catholique de Louvain (UCL), Brussels, 1200, Belgium

**Keywords:** Central sensitization, definition, pain, nociception, secondary hyperalgesia.

## Abstract

Since its discovery, central sensitization has gained enormous popularity. It is widely used to explain pain hypersensitivity in a wide range of clinical pain conditions. However, at present there is no general consensus on the definition of central sensitization. Moreover, the use of the term central sensitization in the clinical domain has been criticized. The aim of this paper is to foster the discussion on the definition of central sensitization and its use.

## Introduction

*“Many subjects, but by no means all, become conscious of soreness of skin surrounding a small area of injury”*

With these words Sir Thomas Lewis starts one of the chapters in his book “Pain” ^1^ (p. 68). The sentence refers to what is now known as “secondary hyperalgesia”, which has intrigued pain neuroscientists for almost a century. Lewis was probably the first that systematically studied this phenomenon. He hypothesized that secondary hyperalgesia was due to a peripheral mechanism (“nocifensor axon reflex”). Impulses generated by nerves at the site of injury travel antidromically via branches to their endings, where there is a release of substances that excite neighboring nerves ^1^.

However, by performing a series of psychophysical experiments Hardy *et al*. ^2^ came to another conclusion. Contrary to Lewis who suggested that secondary hyperalgesia resulted from a spreading of excitation in the skin, Hardy *et al*. hypothesized that secondary hyperalgesia resulted from a “central excitatory state” ^2^ (p. 139).

Similar to the idea of Lewis of a network of interconnected nerves, Hardy *et al*. hypothesized that in the spinal cord there is a pool of neurons consisting of primary and secondary neurons that make synaptic connections to a network of “internuncial” neurons. The function of these internuncial neurons would be to establish and maintain an excitatory state within the neuron pool. In the case of tissue injury, the barrage of noxious impulses originating from the site of injury enters the spinal cord where they excite the network of internuncial neurons, leading to an excitation of connected neurons ^2^.

*“If now the skin is pricked in the area of secondary hyperalgesia, a burst of impulses passes into the spinal cord and when reaching the tertiary neuron it is facilitated giving rise to more intense sensation than usual”* ^2^ (p.135).

Woolf ^3^ was the first that provided evidence for such a “central excitatory state”. He showed that in rats the motor reflex threshold elicited by mechanical punctate stimuli delivered adjacent to a burn injury was reduced for many hours ^3^. In subsequent studies Woolf and co-workers further showed that the induction of this “central excitatory state” does not require tissue injury, but that it can also be induced after electrical stimulation of C-fiber nociceptors ^4^. Based on these findings, Woolf and co-workers ^5^ introduced the term “central sensitization” (CS):

*“This is the phenomenon of aberrant convergence; the generation of pain by activating sensory fibres that normally only produce innocuous sensations i.e. the large myelinated low threshold afferents. Aberrant convergence arises as a consequence of changes induced within the spinal cord by activity in unmyelinated afferent fibres – a process called central sensitization”* (p. 256).

Actually, Woolf *et al*. describe here what is now called allodynia *: “pain in response to a non-nociceptive stimulus”* ^6^.

Since 2008, the task force for taxonomy of the International Association for the Study of Pain (IASP) ^6^ proposes the following definition of CS:

*“Increased responsiveness of nociceptive neurons in the central nervous system to their normal or subthreshold afferent input”*.

The task force for taxonomy ^6^ defines a nociceptive neuron as:

*“A peripheral or central neuron of the somatosensory system that is able to encode a noxious stimulus”.*

But what is meant by *encoding*? And which neurons can be considered part of the somatosensory system and which not?

Nowadays the term CS is very popular and is associated with many more conditions than secondary hyperalgesia. The concept of CS is used by both basic scientists and clinicians; however its use in the clinical domain has been criticized ^7^. The aim of this paper is to foster the discussion on the definition of CS and its use.

## Is CS defined too broadly?

If a definition becomes too broad it will be used non-selectively and it will lose its value. On the other hand, if a definition becomes too specific it may miss important phenomena. The IASP proposal for the definition of CS clearly describes a *phenomenon*. However, in the literature CS is often presented as mechanism, for example, Vardeh *et al*. ^8^ (p. T56). More importantly, the definition does not mention a functional meaning. If the purpose of the term CS was and/or is to explain *pain hypersensitivity* then this should be included in the definition. Furthermore, the term “nociceptive neurons” may then not be specific enough. As pointed out by Sandkühler ^9^:

*“Nociceptive neurons comprise a heterogeneous cell group with putatively many different and sometimes opposing functions, including a large group of inhibitory interneurons. Thus enhanced responsiveness of some of these neurons could contribute to hyperalgesia. On the other hand, enhanced responsiveness of inhibitory nociceptive neurons may well lead to stronger feedback inhibition and analgesia, while still other neurons may not contribute to the experiences of pain but rather to altered motor or vegetative responses to a noxious stimulus”* (p. 708).

Woolf ^10^ proposed an alternative definition of CS which links CS directly to pain hypersensitivity:

*“An amplification of neural signaling within the CNS that elicits pain hypersensitivity”* (p. S5).

However, establishing a causal relationship between CS and pain hypersensitivity is particularly difficult. Indeed, it is possible to measure the activity of nociceptive neurons in the CNS in animal preparations but obviously, we cannot measure pain perception. Conversely, we can measure pain perception in humans but we cannot directly measure the activity of nociceptive neurons ^11^.

In addition, because we cannot record directly from nociceptive neurons in humans and we have to rely on changes in pain perception or thresholds, the risk is to end up in a *circulus in probando* ^12^. For example, patient X shows CS because she/he suffers from pain hypersensitivity and pain hypersensitivity is a manifestation of CS. The described evidence for the conclusion is not different from the conclusion itself.

Taken together, depending on the purpose of the term CS, it may be necessary to reconsider the IASP definition.

## Is secondary hyperalgesia the only example of CS?

In a related note, the task force for taxonomy of the IASP ^6^ further states about the term sensitization:

*“This is a neurophysiological term that can only be applied when both input and output of the neural system under study is known, e.g. by controlling the stimulus and measuring the neural event”*.

According to Treede ^13^ the phenomenon of secondary hyperalgesia induced by intradermal capsaicin injection

“… *is currently the only example where both input and output of spinal neurons have been documented in the same model and, hence, the IASP definition of CS is fulfilled”* (p. 1200).

This would imply that, for the moment, the term CS, as provided by the IASP, may only be used for this particular condition.

When injected into the skin capsaicin activates TRPV1 expressing nociceptors and elicits a burning sensation ^14^. A consequence is the development of increased pinprick sensitivity in a large part of the skin surrounding the injection site ^14^, a phenomenon reminiscent of secondary hyperalgesia after tissue injury. By recording the activity of nociceptive neurons in the primate spinal cord before and after capsaicin injection, Simone *et al*. ^15^ showed that both wide-dynamic-range (WDR) and high-threshold (HT) neurons respond more strongly to pinprick stimuli when these stimuli were delivered after the injection to the skin surrounding the injection site (output). The same group also recorded the activity of peripheral A-fiber and C-fiber nociceptors in this area (input) but their activity was unchanged ^16^. Because these sensitized spinal neurons project via the spinothalamic pathway to the brain, they may contribute to the increase in pinprick perception in humans.

However, it remains puzzling why secondary hyperalgesia is characterized by an increase in the perception for mechanical pinprick stimuli, but not heat stimuli ^17– 19^. Should a sensitization of WDR neurons, which are polymodal, not also lead to an increase in perception for other modalities like touch or heat?

## Nociceptive input (and increases thereof) does not necessarily elicits pain

An important function of nociception in normal conditions is to warn for tissue damage. Therefore it would make sense that nociceptors are activated *before* there is any tissue damage. Compatible with this idea are the observations that nociceptors in humans are activated by stimulus intensities that are *not perceived as painful* ^20^.

Indeed, in normal conditions (i.e. without sensitization) mechanical pinprick stimuli typically elicit a sharp pricking sensation, which is not perceived as painful in the majority of people. However, studies using microneurography have clearly demonstrated that such mechanical pinprick probes activate mechanosensitive nociceptors in the skin ^21– 23^. Moreover, a study comparing the perceptual pain thresholds in human volunteers with the thresholds for nociceptors in animals using the same pinprick probes, suggests that the non-painful sharp pricking sensation is mediated by mechanosensitive nociceptors ^24^.

Pinprick stimuli delivered after sensitization to the skin surrounding the site at which sensitization was induced clearly elicit an increase in intensity of perception but this is not always perceived as painful. Importantly, the perception elicited by tactile stimuli is not increased ^25^ (and unpublished observations), indicating that the increase in the pricking sensation elicited by pinprick stimuli after sensitization is mediated by mechano-sensitive nociceptors instead of low-threshold mechanoreceptors.

Likewise, we recently showed that heat perception elicited by tiny laser stimuli selectively activating C-fiber nociceptors in the skin was greater when these stimuli were delivered to the area of secondary hyperalgesia ^26^. However, despite the fact that our heat stimuli selectively activated C-fiber nociceptors, the perception elicited by these stimuli was not qualified as painful neither at baseline (before inducing sensitization) nor after the induction of sensitization. Importantly, the greater heat sensitivity elicited by these stimuli is probably a perceptual correlate of CS. Indeed, Kronschläger *et al*. ^27^ recently showed in rats that strong peripheral nociceptive input activates glial cells (which include microglial and astrocytes) leading to the release of cytokines and chemokines which excites remote C-fiber synapses.

Taken together, both examples (increased pinprick sensitivity and greater heat sensitivity) suggest that CS does not necessarily result in pain hypersensitivity. This would plead for a mechanism-based approach of CS rather than focusing on changes in pain perception only. Indeed, according to the definitions provided by the IASP ^6^ one cannot label the increases in pinprick and heat perception as “hyperalgesia” because it is not an *increase in pain sensitivity*. They cannot be labeled as “allodynia” either, because the stimulus is a nociceptive one and is not always perceived as painful after sensitization.
