# Supplementary material for: Central sensitization and pain hypersensitivity: Some critical considerations
Source: F1000Res. 2018 Aug 31;7:1325. Originally published 2018 Aug 21. [Version 2] doi: 10.12688/f1000research.15956.2 (PMC6206600; doi:10.12688/f1000research.15956.2)
Supplement: Peer review history

# Peer review: Referee response for version 2

**DOI:** 10.5256/f1000research.17668.r38593

**Published:** 2018-10-23

**Authors:** Philipp Hüllemann

**Affiliations:** 1 Division of Neurological Pain Research and Therapy, Department of Neurology, University of Kiel, Kiel, Germany

Emanuel N van den Broeke provides a very interesting overview on how the term ‘central sensitization’ (CS) was originally characterized, how the use of the term developed in the scientific field and how extensively it may now be overused in basic and clinical research. The author lists several “historic” and recent scientific examples, which shine light on the mechanistic origin of central sensitization. It soon becomes clear that there is no actual consensus on the definition of central sensitization and that scientific evidence is sparse as well as contradictory on some occasions. Newer studies show that the intensity of thermal and mechanical stimuli increases most probably due to central sensitization processes but that this increase of intensity is not necessarily perceived as painful. Therefore, non-painful aspects of central sensitization are lacking in the current definition of CS. The further, we need to think of a more specific definition, which may guide researchers and clinicians in the use of the term.

 I have two suggestions:

  

1. It might have been useful to add some sentences on peripheral sensitization and its possible role in driving, as well as maintaining central sensitization.
2. A short conclusion/summary including the authors thoughts would also be helpful.

I have read this submission. I believe that I have an appropriate level of expertise to confirm that it is of an acceptable scientific standard.

# Peer review: Referee response for version 2

**DOI:** 10.5256/f1000research.17668.r38228

**Published:** 2018-10-18

**Authors:** Geert Crombez

**Affiliations:** 1 Department of Experimental-Clinical and Health Psychology, Ghent University, Ghent, Belgium

The paper by Emmanuel van den Broeke critically discusses definition and use of the terms central sensitisation and pain hypersensitivity. As stated by the author, the term central sensitisation has become increasingly popular, and seems to become over-used, if not mis-used. At times, it is important to reflect upon the origin of terms, and to track how meaning and use have changed over time. Evidently, we do not have to hold on the past, and definitions and use may change as science advances. The paper of van den Broeke is timely, and provides essential reading for many. It is an ideal paper for scholarly reflection and group discussion.

 It nicely traced the origin in meaning, and the various changes in definition. It critically analyses interrelationships with other constructs, and potential disadvantages. Notwithstanding, it does not provide definite answers. Probably, that is not possible, but I would suggest that the authors reflect upon what should be the way forward. What do they recommend to readers and researchers.

 Most importantly, seems to be a precise use of the term, and to avoid confusion in meaning. Indeed, as pointed out central sensitisation can be used to describe a phenomenon or to describe a mechanism. This is confusing and may result in circular reasoning: central sensitisation explains central sensitisation; In that respect, I have learned to make a distinction between at least three ways of using scientific terms: (1) as a result, (2) as an explanation and (3) as a procedure. Central sensitisation as a result refers to the phenomenon, most often as the result of a specific procedure. Indeed, there are some experimental procedures that induce the phenomenon. Finally, an scientific endeavour is to provide explanations, often mechanistic explanations, for the phenomenon that results from particular experimental procedures. In times of confusion and overuse, it is useful to come back and reflect upon what exactly is meant by someone.

I have read this submission. I believe that I have an appropriate level of expertise to confirm that it is of an acceptable scientific standard.
